# Discovery of a diagnostic biomarker for colon cancer through proteomic profiling of small extracellular vesicles

**DOI:** 10.1186/s12885-018-4952-y

**Published:** 2018-11-01

**Authors:** Chan-Hyeong Lee, Eun-Ju Im, Pyong-Gon Moon, Moon-Chang Baek

**Affiliations:** 1 Department of Molecular Medicine, CMRI, School of Medicine, Kyungpook National University, Daegu, 41944 Republic of Korea; 2 Exosome Convergence Research Center (ECRC), School of Medicine, Kyungpook National University, Daegu, 41944 Republic of Korea

**Keywords:** Small extracellular vesicle, Proteomics, Colon cancer, Biomarker, Diagnosis

## Abstract

**Background:**

Small extracellular vesicles (small-EVs) are membranous vesicles that contain unique information regarding the condition of cells and contribute to the recruitment and reprogramming of components associated with the tumor environment. Therefore, many researchers have suggested that small-EV proteins are potential biomarkers for diseases such as cancer. Colon cancer (CC) is one of the most common causes of cancer-related deaths worldwide. Biomarkers such as carcinoembryonic antigen (CEA) show low sensitivity (~ 40%), and thus the demand for novel biomarkers for CC diagnosis is increasing.

**Methods:**

In this study, we identified biomarkers for diagnosing CC through proteomic analysis of small-EVs from CC cell lines. These small-EVs were characterized by western blot analysis, nanoparticle tracking analysis, and transmission electron microscopy and analyzed using mass spectrometry.

**Results:**

Five selected proteins were found to be upregulated in CC by western blot analysis. Among the candidate proteins, tetraspanin 1 (TSPAN1) was found to be upregulated in plasma EVs from CC patients compared to those from healthy controls (HCs) with 75.7% sensitivity.

**Conclusions:**

These results suggest that TSPAN1 is a potent non-invasive biomarker for CC detection. Our experimental strategy provides useful insights into the identification of cancer-specific non-invasive biomarkers.

**Electronic supplementary material:**

The online version of this article (10.1186/s12885-018-4952-y) contains supplementary material, which is available to authorized users.

## Background

Colon cancer (CC) is the third most common cause of cancer-related deaths worldwide and its mortality and incidence rates are increasing rapidly [1]. This disease mainly occurs in males and females 55–85 years of age; this age group accounts for approximately 80% of CC cases. Common risk factors for CC include obesity, family history, and physical inactivity [2–4]. CC is typically diagnosed by endoscopic biopsy and can be carried out by either sigmoidoscopy (as > 35% of tumors are in the rectosigmoid) or (preferably) total colonoscopy. [5]. However, these methods have limitations in terms of their convenience, risk, cost, and sensitivity [6]. Blood-based biomarkers can be analyzed easily, quickly, and economically, and therefore have the potential to greatly improve diagnostic efficiency. In 1965, Dr. Joseph Gold discovered a protein that is normally found in the gastrointestinal tissue during fetal development in the blood of patients with colon cancer, which was named as carcinoembryonic antigen (CEA). This protein is now used as a biomarker for the diagnosis and prognosis of CC in hospitals [7]. However, most biomarkers for other cancers, including CEA, show limited sensitivity [8]. Thus, novel diagnostic biomarkers with high specificity and sensitivity are required for diagnosing CC.

Extracellular vesicles (EVs) are membrane-bound vesicles secreted by a variety of cell types and are found in various body fluids including the blood, urine, breast milk, and malignant ascites [9]. These vesicles also contain oncogenic proteins [9], signaling molecules [10], lipids [11], mRNAs, and microRNAs [12] that reflect parental cell functions [13], and can be horizontally transferred to recipient cells to regulate their characterization [12]. Among these, small extracellular vesicles (small-EVs), including exosomes, have been studied in various cancer types [13] and small-EV cargo contains more oncoproteins than large EVs [14].

Cancer cells secrete small-EVs containing oncogenic molecules into the extracellular environment, which play important roles in metastasis, angiogenesis, cancer cell proliferation, modulation of the tumor microenvironment, and immune suppression [15]. It has been suggested that cancer cell-derived small-EVs can be used as biomarkers for the diagnosis and prognosis of various cancers [16–18].

Recently, small-EVs isolated from cancer cells have been used to identify novel cancer-related biomarkers. Several studies were performed to identify molecular biomarkers in cancer cell-derived small-EVs [18–20]. However, most methods require a long time and large sample volume for small-EV isolation. To overcome these problems, we developed an enzyme-linked immunosorbent assay (ELISA) method to detect small-EV proteins without isolating the small-EVs. We examined whether this method can be used to diagnose early breast cancer; surface proteins (Del-1 and fibronectin) in small-EVs were detected in plasma obtained from patients with breast cancer with high sensitivity and specificity [21, 22]. The results suggest that an optimized ELISA method can detect surface proteins in small-EVs and that small-EV proteins can effectively distinguish between HCs and cancer patients.

In this study, we aimed to discover potential biomarkers in small-EVs through proteomic analysis for diagnosing CC and identified small-EVs from plasma for distinguishing between healthy controls (HCs) and CC patients without the need of an isolation step for small-EVs.

## Methods

### Cell lines and cell culture

HT-29 (ATCC® Number HTB-38™), HCT-116 (ATCC® Number CCL-247™) CC epithelial cells and CRL-1541 (ATCC® CRL-1541™) colon normal fibroblasts were cultured in Dulbecco’s modified Eagle’s medium (DMEM) supplemented with 10% EV-depleted fetal bovine serum (FBS) and 1% antibiotic/antimycotic solution. Cells were obtained from the American Type Culture Collection (Manassas, VA, USA) and tested for mycoplasma contamination by PCR.

### Cell proliferation assay

HT-29 and HCT-116 cells (50,000 cells/well) were seeded into 24-well plates in DMEM supplemented with 10% EV-depleted FBS and cultured to 90% confluence. The medium was replaced with serum-free medium after 0, 12, 24, and 48 h, and 0.5 mg/mL MTT (3-(4,5-dimethylthiazol-2yl)-2,5-diphenyltetrazolium bromide) solution was added to each well and incubated for 3 h at 37 °C. Five hundred microliters of 100% isopropanol was added, 200 μL of this mixture was transferred to 96-well plates, and absorbance was measured at 495 nm.

### Small-EV purification

Small-EVs were purified by differential centrifugation as described previously [23, 24]. Briefly, supernatants from CC cells were subjected to differential centrifugation at 300, 1500, 2500, and 100,000×*g*. Small-EVs (100,000×*g* pellet) were resuspended in phosphate-buffered saline (PBS) for further analysis. As an alternative method for purification of small-EVs from plasma, the plasma was mixed with ExoQuick (System Biosciences, Mountain View, CA, USA). An optimized ExoQuick method was used as described in our previous study [25]. In the optimized ExoQuick method, after incubation at 4 °C for 30 min, the samples were centrifuged at 1500×*g* for 30 min, and the pellet was washed three times with PBS. These extra steps were required to reduce contamination with plasma proteins such as albumin. Small-EV proteins were quantified using the Pierce BCA Protein Assay kit (Rockford, IL, USA) after treatment with RIPA buffer (Cell Signaling Technology, Danvers, MA, USA).

### Transmission electron microscopy (TEM)

Purified small-EVs were deposited onto pure carbon-coated EM grids. After staining with 2% uranyl acetate, the grids were dried at 25 °C and visualized at 6000× and 10,000× magnification using the Hitachi H-7000 transmission electron microscope (Tokyo, Japan).

### Nanoparticle tracking analysis (NTA)

The number of small-EVs was measured by NTA as described in our previous study [26]. Suspensions containing small-EVs from plasma or cell culture medium were analyzed using the Nano-Sight LM10 instrument (NanoSight, Wiltshire, UK). For this analysis, a monochromatic laser beam (405 nm) was applied to a dilute suspension of small-EVs. A video of 30- to 60-s duration was recorded at a rate of 30 frames/s, and small-EV movement was analyzed using NTA software version 2.2 (NanoSight). NTA post-acquisition settings were optimized and remained constant between samples, and each video was analyzed to estimate the concentration.

### Gel-assisted digestion

Protein concentrations of small-EVs in HT-29, HCT-116, and CRL-1541 cells were determined using the BCA assay after cell lysis. Digestion was performed as described previously [24]. Briefly, 50 μg protein was resuspended in 100 mM triethylammonium bicarbonate (TEABC; pH 8) containing 6 M urea, 5 mM EDTA, and 2% SDS. The proteins were chemically denatured by adding 10 mM dithiothreitol and incubating at 60 °C for 20 min. Next, proteins were alkylated by adding 50 mM iodoacetamide at room temperature and incubating for 20 min. The protein solution was mixed with acrylamide/bisacrylamide solution (30% *v*/v, 29:1) containing 10% (*w*/*v*) ammonium persulfate and tetramethylethylenediamine. The gel was cut into small pieces and washed three times with 50 mM TEABC containing 50% acetonitrile (ACN). Proteolytic digestion was performed using trypsin (protein:trypsin = 50:1 *w*/w) in 50 mM TEABC and incubating overnight at 37 °C. The digested peptides were extracted from the gel by exchanging with two buffers: 0.1% formic acid (FA) in 50 mM TEABC and 0.1% FA in 50% ACN. The eluents were concentrated using a Speedvac and desalted using an HLB cartridge (Waters, Milford, MA, USA).

### Liquid chromatography-tandem mass spectrometry (LC–MS/MS) analysis

Small-EVs were analyzed by LC–MS/MS as described previously [27]. One microgram of extracted peptide was analyzed by nano-ultra-high-performance LC (UPLC) (Waters) and tandem mass spectrometry using a Q-Tof Premier (Waters). Peptides were injected into a 2 cm × 180 μm trap column and resolved in a 25 cm × 75 μm nanoACQUITY C18 column (Waters) using the LC system. Mobile phase A was composed of water containing 0.1% FA and mobile B phase was 0.1% FA in ACN. The peptides were resolved using a gradient of 3–45% mobile phase B over 135 min at a flow rate of 300 nL/min. All samples were analyzed in triplicate. The method included a full sequential MS scan (m/z 150–1600, 0.6 s) and five MS/MS scans (m/z 100–1990, 0.6 s/scan) for the five most intense ions present in the full-scan mass spectrum.

### Proteomic data processing and analysis

For protein identification, MS raw data were converted into peak lists using MASCOT Distiller version 2.1 (Matrix Science, London, UK) with default settings. All MS/MS raw data were analyzed using MASCOT version 2.2.1 (Matrix Science) [27]. Mascot was used to search the Swiss-Prot database (release 2018_07) with human taxonomy. The database search against Mascot was performed with a fragment ion mass tolerance of 0.5 Da and parent ion tolerance of 0.2 Da. Two missed cleavages were allowed for trypsin digestion. Carbamidomethylation of cysteine residues and oxidation of methionine residues were considered as variable modifications. To evaluate the false discovery rate (FDR) of protein identification, this analysis was repeated using identical search parameters and validation criteria against a randomized decoy database created by Mascot. Peptide identities were assigned if their Mascot ion scores corresponded to *p* < 0.05. Proteins with more than 2 peptides were identified with confidence. FDRs with Mascot protein scores > 34 (*p* < 0.05) ranged below 2%.

### Human plasma collection

Human blood samples were obtained from 33 healthy donors and 46 patients with CC at Kyungpook National University Hospital (KNUH) and Chonbuk National University Hospital. All individuals provided informed consent for blood donation according to a protocol approved by the Institutional Review Board of KNUH. The blood samples of subjects from both groups were collected preoperatively in 9-mL vacutainers containing EDTA. The collected blood samples were centrifuged at 1500×*g* for 15 min to obtain plasma. These supernatants were stored at − 80 °C until use.

### Immunoblotting

Proteins were resolved by SDS-PAGE, transferred onto nitrocellulose membranes, probed with each primary antibody, and incubated with horseradish peroxidase-conjugated secondary antibody. The blots were visualized with enhanced chemiluminescence detection reagents (Thermo Fisher Scientific, Waltham, MA, USA) and quantified using enhanced chemiluminescence hyperfilm (AGFA, Morstel, Belgium). The following primary antibodies were used against CD63 (ab68418, 1:1000), HSP70 (ab2787, 1:1000), CD9 (ab92726, 1:1000), Alix (ab56932, 1:1000), LGALS3BP (ab123921, 1:1000), SLC1A5 (ab58690, 1:1000), CLDN7 (ab53044, 1:1000), RAI3 (ab155557, 1:1000; all from Abcam, Cambridge, UK), and TSPAN1 (H00010103, 1:1000; Abnova, Taipei City, Taiwan).

### Elisa

The method for detecting small-EV proteins has been described previously [21]. Anti-CD63 antibody (1:250)-coated 96-well plates were blocked with PBS containing 0.05% Tween 20 (PBS-T) for 3 h at room temperature. Serial dilutions of mouse serum were prepared in PBS-T supplemented with 10% horse serum. The diluted solution was added to the plates in duplicates and incubated for 2 h at room temperature. After washing, the samples in the plates were reacted with the monoclonal anti-TSPAN1 antibody pre-incubated with a horseradish peroxidase-linked secondary antibody for 30 min and developed using 3,3′,5,5′-tetramethylbenzidine-containing peroxide. The reaction was stopped, and optical density values were measured at 450 nm using an automated iMark (Bio-Rad, Hercules, CA, USA).

### Statistical analysis

Data are presented as the mean ± SD, as indicated in each graph. The Student’s *t*-test was used to evaluate the differences between means for normally distributed immunoblotting data. The significance of the ELISA results was analyzed using the Mann-Whitney test. All *p*-values were two-tailed, and *p*-values less than 0.05 were considered statistically significant. Receiver operating characteristic (ROC) curves were generated based on logistic regression from the ELISA data. Statistical analysis was performed using SPSS 22.0 for Windows (SPSS, Inc., Chicago, IL, USA). The area under ROC curve (AUC) calculation provided a convenient number. AUC < 0.5 indicated that the test showed no difference between the two groups, while 0.5 < AUC < 1.0 indicated that the test yielded perfect differentiation between groups at *p < 0.05, **p < 0.01, ***p < 0.001, or ****p < 0.0001.

## Results

### Isolation and characterization of small-EVs in colon cancer cell lines

**Fig. 1:**
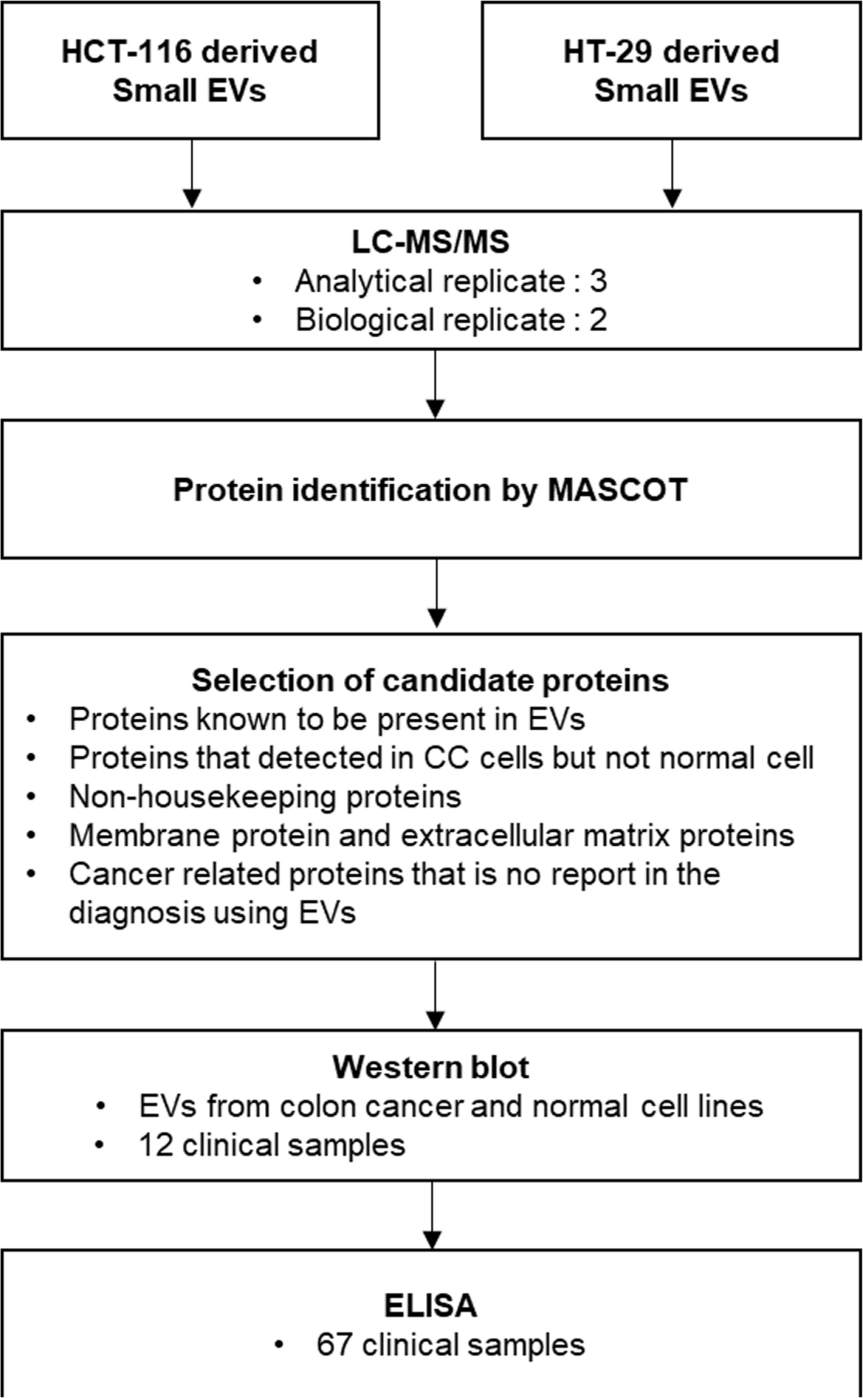
Schematic representation of the workflow. Small-EV proteins from HT-29 and HCT-116 colon cancer cell lines were identified by proteomic analysis and candidate proteins predicted to be upregulated in colon cancer patients compared to the healthy controls were selected. Candidate proteins were verified by western blot analysis. Among these candidates, TSPAN1 was further validated by ELISA

To identify potential biomarkers for diagnosing colon cancer, HT-29 and HCT-116 human colon cancer cell lines were used in this study. A schematic workflow is shown in Fig. 1.

**Fig. 2:**
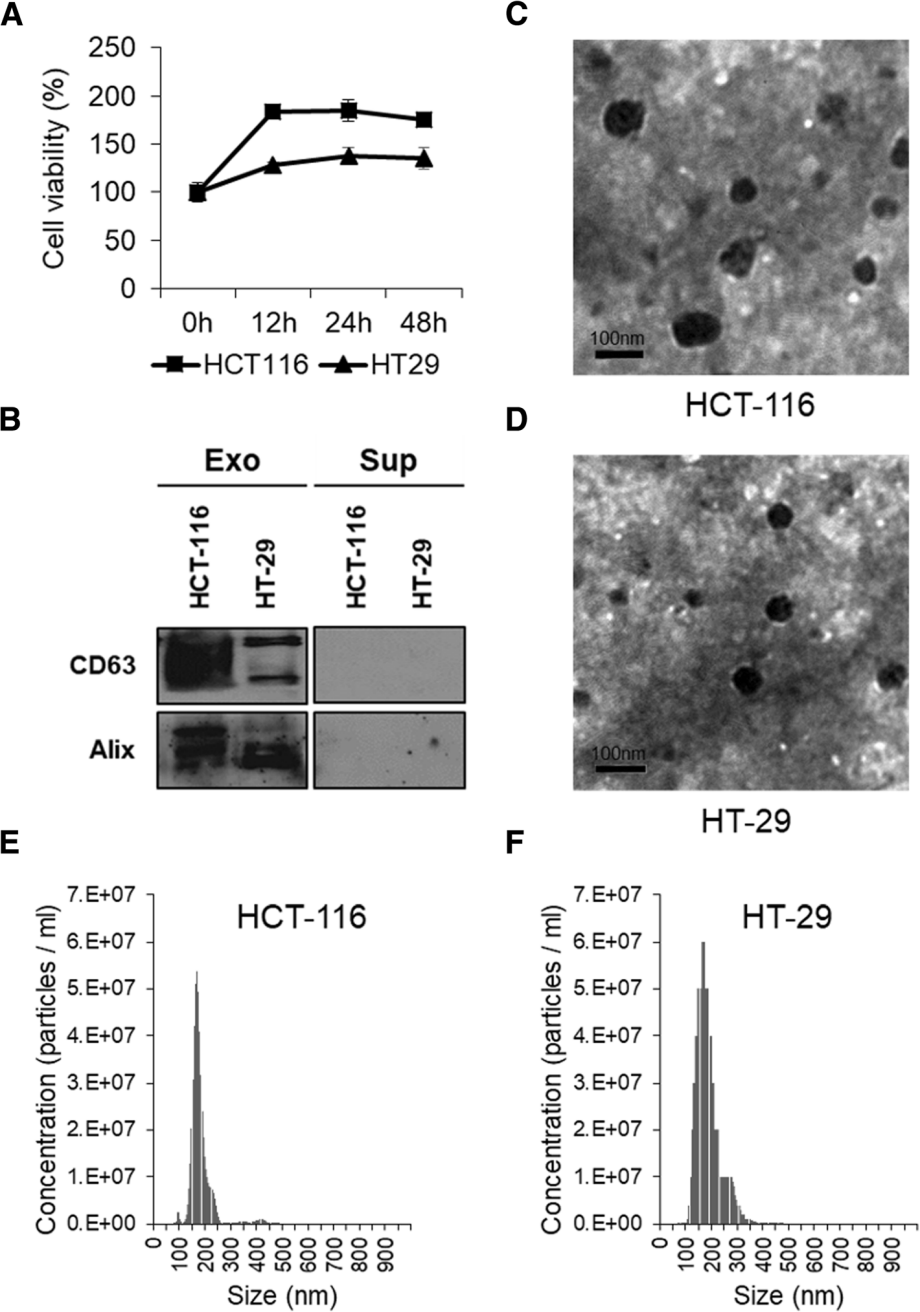
Characterization of small-EVs isolated from HT-29 and HCT-116 colon cancer cells. **a** Viability of HT-29 and HCT-116 colon cancer cells following exposure to serum-free medium was measured using the MTT assay. **b** Small-EVs (10 μg protein) from HT-29 and HCT-116 cells were subjected to immunoblotting with antibodies against small-EV marker proteins, namely CD63 and Alix. **c**, **d** Small-EVs derived from HT-29 and HCT-116 cells were analyzed by transmission electron microscopy. All small-EV samples contained vesicles of variable sizes in the range of 70–200 nm. Scale bar, 100 nm. **e**, **f** Size distribution of small-EVs derived from two different colon cancer cell lines was measured using nanoparticle tracking analysis. The images shown are representative and the size of HT-29- and HCT-116-derived small-EVs is 184.2 ± 46.5 and 178.5 ± 44.4 nm, respectively (*n* = 3)

We analyzed cell viability at different incubation times under starvation conditions to optimize the incubation time and observed reduced cell viability at more than 24 h incubation (Fig. 2a). Isolated small-EVs were analyzed by western blotting, TEM, and NanoSight. Immunoblotting revealed that the small-EV fraction was highly enriched in small-EV markers such as CD63 and Alix (Fig. 2b). It also showed rounded morphology (Fig. 2c) and a size distribution (Fig. 2d) consistent with typical small-EVs.

### Identification of small-EVs from CC cells by proteomic analysis

**Fig. 3:**
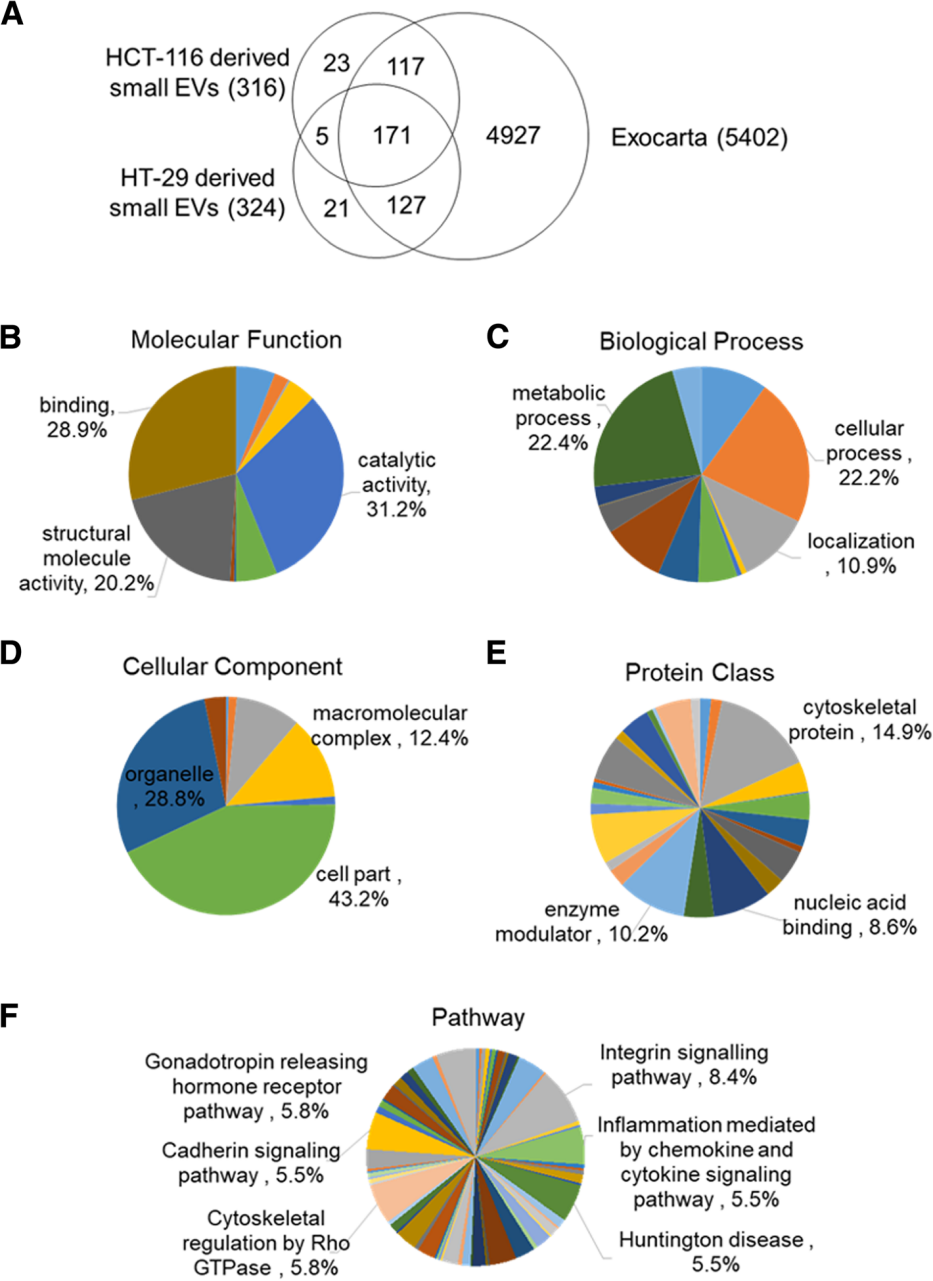
Proteomic analysis of small-EVs from HCT-116 and HT-29 colon cancer cells. Search results were identified using MASCOT software. **a** At the cut-off settings, 464 human proteins were identified; of these, 176 proteins were observed to be common between the two cell lines. In total, 140 proteins were uniquely identified in the HCT-116-derived small-EVs and 148 proteins were identified in the HT-29-derived small-EVs. Among these, 415 proteins were present in the ExoCarta database. Identified proteins were segregated into various groups using PANTHER software. Gene ontology analysis for molecular function **b**, biological process **c**, cellular component **d**, protein class **e**, and pathway **f** of the proteins identified in small-EVs was performed using PANTHER software

We performed proteomic analysis of small-EVs from CC cell lines by MS to identify small-EV proteins. CC cell-derived small-EVs were analyzed in two biological and three analytical replicates using nano-UPLC–MS/MS. Small-EV proteins were searched in the Swiss-Prot database with human taxonomy using the MASCOT software. From among high-confidence peptide sequences with error rates < 5%, we identified 316 and 324 proteins in HCT-116- and HT-29-derived small-EVs, respectively. This analysis showed that proteins with a peptide hit score (PHS) > 1 were identified with high confidence by multiple peptides and overlapping gene names were removed. A total of 464 proteins were identified in CC cell-derived small-EVs with high confidence (PHS > 1) (Fig. 3a; see Additional file 1: Table S1). Among these, 176 proteins were common between the two cell lines. We compared the identified proteins with the data in ExoCarta and observed that 90% of the identified small-EV proteins were present in this database (Fig. 3a). For bioinformatics analysis, including prediction of the function, biological process, cellular components, localization, and pathways of the exosomal proteins, we used the Protein Analysis Through Evolutionary and Relationship (PANTHER) software. The identified proteins were associated with at least one annotation term. All searched proteins were categorized as having binding (36.3%), catalytic (32.2%), or structural (13.6%) activities (Fig. 3b). The distribution of biological processes was as follows: cellular (29.1%), metabolic (19%), and cellular component organization or biogenesis (12.2%) (Fig. 3c). As shown in cellular components, 35.9% proteins were predicted to be localized in the cytoplasm, 24.5% in organelles, and 17.5% in macromolecular complexes (Fig. 3d). The distribution by protein class was as follows: enzyme modulator (15.9%), nucleic acid-binding (13.2%), and cytoskeletal protein (12.1%) (Fig. 3e). Signaling pathway analysis of the identified proteins confirmed their associations with a variety of pathways (Fig. 3f).

### Selection of candidate proteins for diagnosis of colon cancer

From the identified proteins, we selected candidate proteins to identify CC-specific proteins. The filtration step to select the candidate proteins is shown in Fig. 1. A total of 464 small-EV proteins were identified in the HCT-116 and HT-29 cell-derived small-EVs by mass spectrometry. Among these, 415 proteins known to be present in EVs were selected. Proteins detected in HCT-116- and HT-29-derived small-EVs, but not in small-EVs from CRL-1541 or normal colon cells (see Additional file 2: Table S2), were selected (352). Next, we excluded housekeeping proteins such as actin, myosin, and ribosomal proteins (231). Extracellular matrix proteins and outer membrane proteins were selected to detect small-EVs in the plasma without a purification step (114). Finally, we selected the following five proteins based on previous studies: TSPAN1, galectin-3-binding protein (LGALS3BP), neutral amino acid transporter B(0) (SLC1A5), claudin 7 (CLDN7), and retinoic acid-induced protein 3 (GPRC5A).

### Expression of candidate proteins in HT-29- and HCT-116-derived small-EVs

**Fig. 4:**
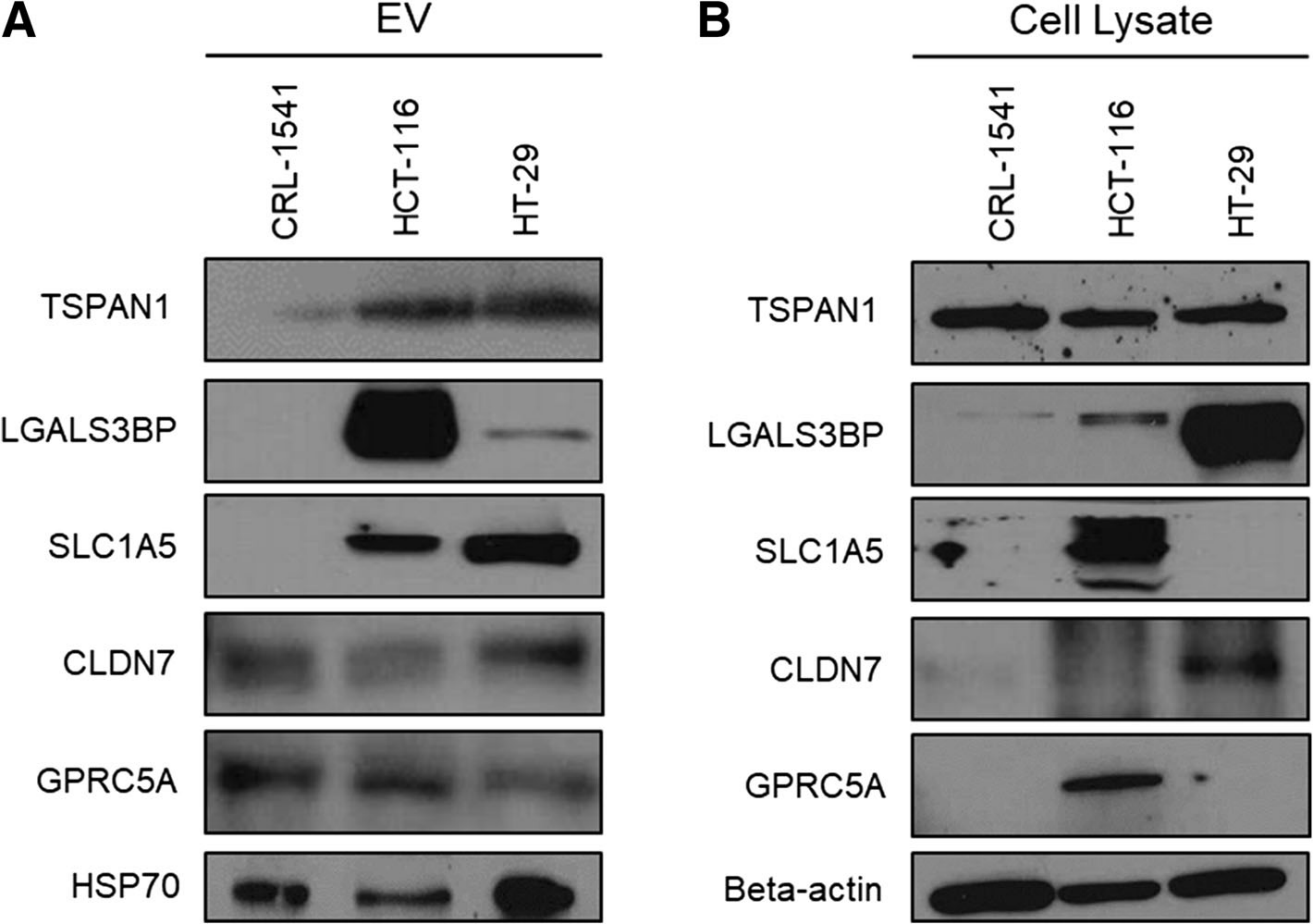
Immunoblot showing candidate proteins from small-EVs and cell lysates of colon cancer and normal cells. The expression of selected proteins, namely TSPAN1, LGALS3BP, SLC1A5, CLDN7, and GPRC5A, in small-EVs was validated. Protein samples were prepared from small-EVs (10 μg protein) (**a**) and cell lysates (10 μg protein) (**b**) of HT-29, HCT-116, and CRL-1541 cell lines

We performed immunoblotting analysis to detect candidate proteins in CC cells and confirm whether these proteins observed in the proteomic analysis were truly expressed in the small-EVs isolated from CC cells. CRL-1541 cells were analyzed and compared with CC cells. HSP70 was used as a known small-EV marker and β-actin was used as a cytosol marker for cell lysates. All candidate proteins were distinctly detected in CC cell-derived small-EVs (Fig. 4). Among these, expression of three proteins (SLC1A5, LGALS3BP, and TSPAN1) was increased in small-EVs isolated from CC cell lines.

### Expression of candidate proteins in the plasma of CC patients analyzed by immunoblotting assay

**Fig. 5:**
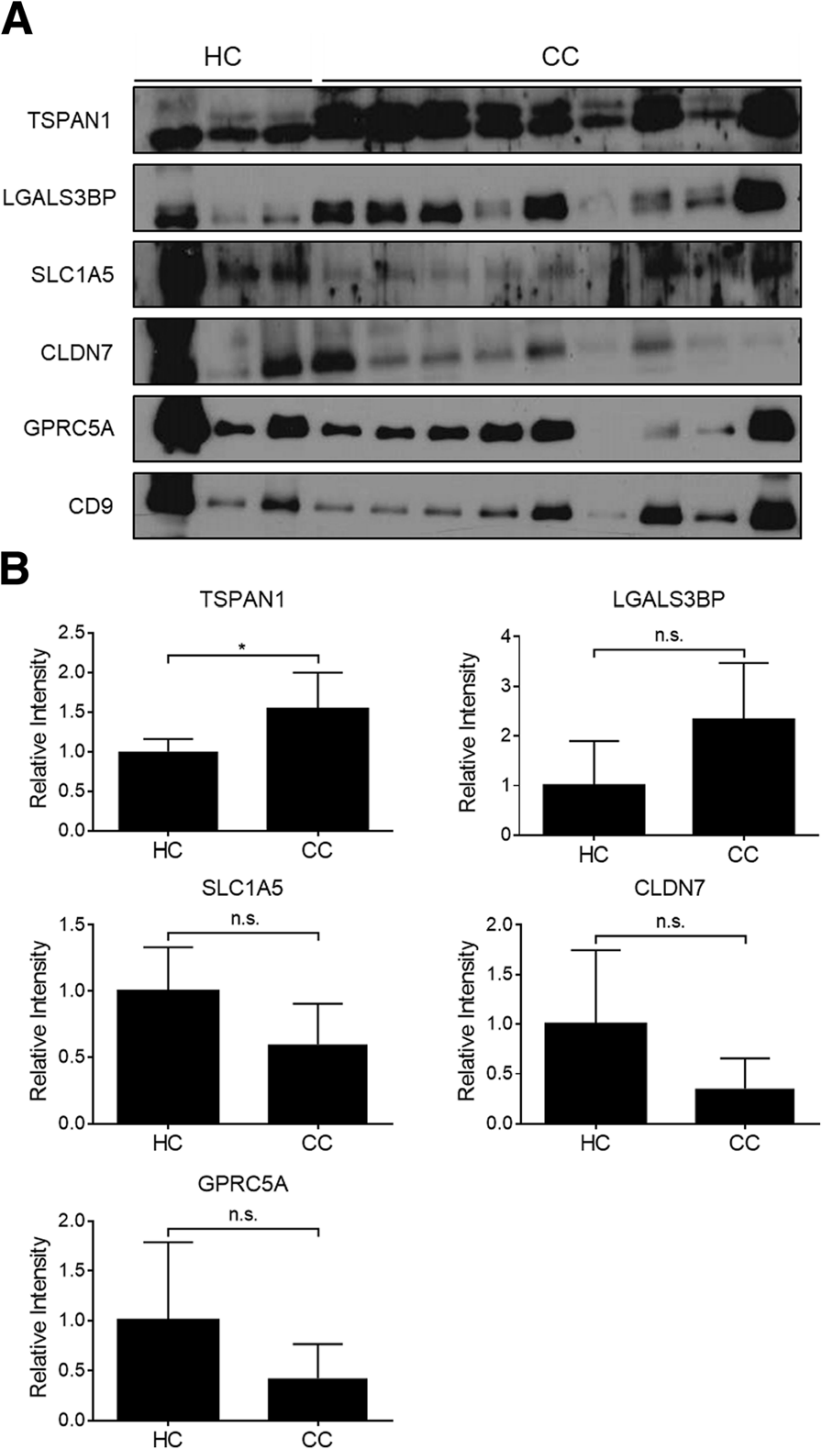
Expression of candidate proteins in plasma-derived small-EVs. **a** Western blotting was performed to determine the expression level of five potential colon cancer markers and CD9 (a positive-control small-EV marker) in plasma-derived small-EVs (4 μL) obtained from colon cancer patients (CC, *n* = 9) and healthy controls (HC, *n* = 3). **b** Expression level and significance were validated. Significance was analyzed using the Student’s *t*-test. *p < 0.05

To verify the levels of selected proteins in clinical samples, we used human plasma from HCs and patients with colon cancer. Detailed data related to clinical sample characteristics are provided in Table 1. After isolating small-EVs from the plasma, the selected proteins in small-EVs isolated from HC (*n* = 3) and CC patients (*n* = 9) (Fig. 5a) were verified by immunoblotting. Relative expression of the candidate biomarker proteins is shown in Fig. 5b in HC vs CC patients. TSPAN1 expression (HC:CC = 1:1.5, *p* = 0.013) was significantly higher in small-EVs isolated from the plasma of CC patients than in those of HC patients; however, LGALS3BP (HC:CC = 1:2.3, *p* > 0.05), SLC1A5 (HC:CC = 1:0.6, *p* > 0.05), CLDN7 (HC:CC = 1:0.4, *p* > 0.05), and GPRC5A (HC:CC = 1:0.4, *p* > 0.05) expression was not significantly different between the two groups (Fig. 5b).

**Table 1:** Information about clinical samples from patients with colon cancer and healthy controls

Detection method		Western blotting				ELISA			
Clinical information		# of samples (*n* = 12)	Age	Sex		# of samples (*n* = 67)	Age	Sex	
				M	F			M	F
Healthy controls		3	46.3 ± 10.9	2	1	30	50.9 ± 12.9	15	15
Colon cancer patients	Stage 1	3	66 ± 10.4	2	1	10	68.7 ± 10.8	5	5
	Stage 2	3	64 ± 4.6	1	2	10	64 ± 9.1	5	5
	Stage 3	3	63.7 ± 3.8	2	1	10	67.4 ± 7.1	5	5
	Stage 4					7	63.4 ± 12.4	4	3

### Diagnostic value of TSPAN1-positive small-EVs in plasma from CC patients analyzed using ELISA

**Fig. 6:**
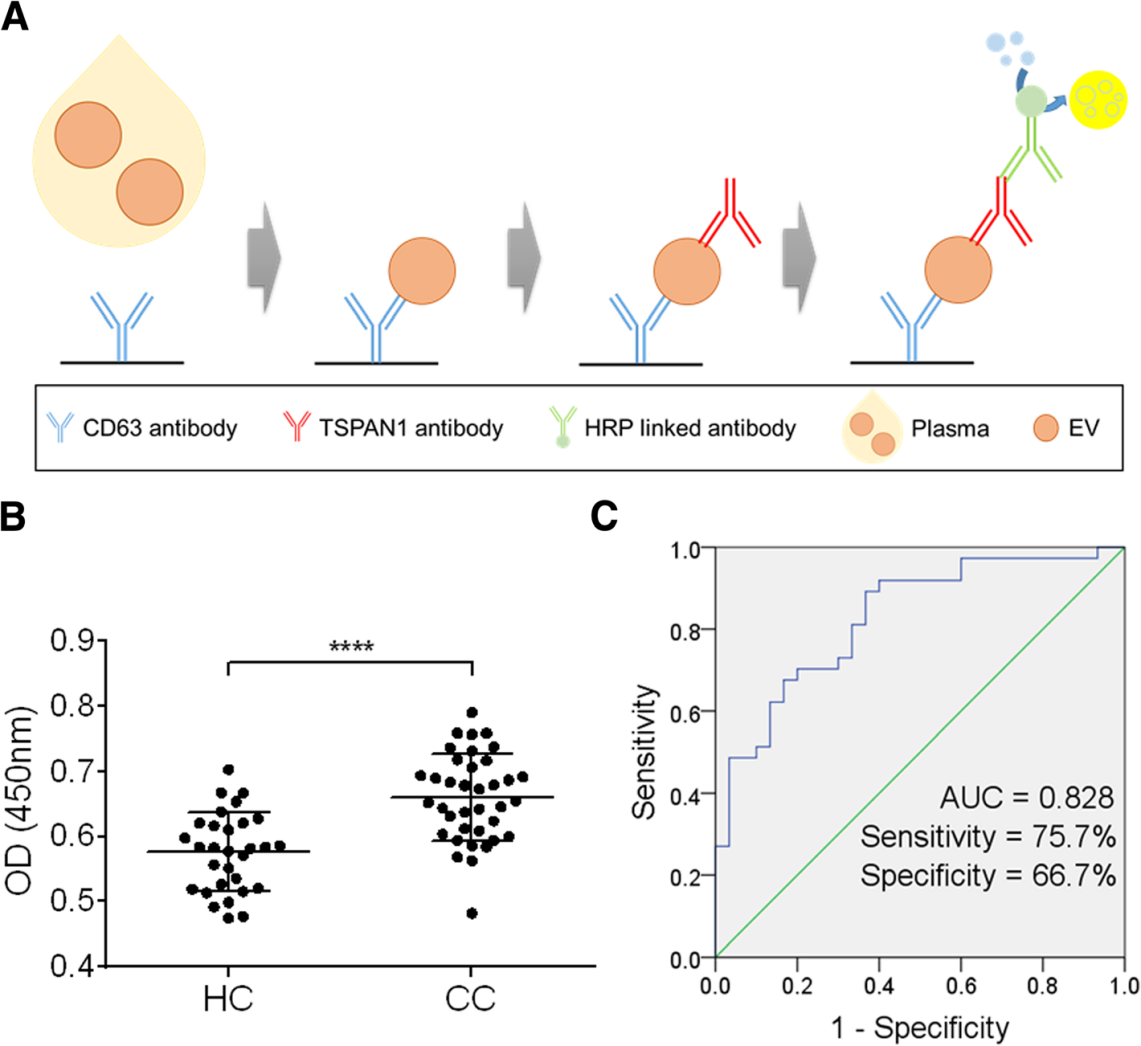
Diagnostic value of TSPAN1-positive EVs in plasma. **a** TSPAN1-positive small-EVs in plasma were captured using the coated anti-CD63 antibody and detected using the anti-TSPAN1 antibody. **b** TSPAN1-positive small-EVs were validated in the plasma of colon cancer patients (CC, *n* = 37) and healthy controls (HC, *n* = 30). Significance was analyzed using the Mann-Whitney test. ****p < 0.0001. **c** ROCs for differentiating between CC patients and HCs were analyzed. The AUC, sensitivity, and specificity for TSPAN1 are shown

We validated TSPAN1 in small-EVs from HCs (*n* = 30) and CC patients (*n* = 37) by ELISA. TSPAN1-positive small-EVs in plasma were captured using a coated anti-CD63 antibody and detected using an anti-TSPAN1 antibody (Fig. 6a). Our previous studies showed that this method was effective for detecting small-EVs [31, 32]. These results indicated that the TSPAN1 level was significantly higher in CC patients than in HCs (Fig. 6b). Additionally, ROC curves were generated using ELISA results to describe diagnostic performance (Fig. 6c), and AUC was determined for TSPAN1. Fig. 6 shows that the AUC of TSPAN1 for differentiating between CC patients and HCs was 0.828 (95% CI = 0.73–0.926), with a sensitivity of 75.7% and specificity of 66.7%.

## Discussion

Small-EVs are informative vesicles that reflect the parent cell’s physiological state and contain nucleic acids and proteins. Therefore, we focused on identifying biomarkers for diagnosing CC by analyzing small-EV proteins. In this study, we investigated cancer-related proteins from HCT-116 and HT-29 CC cell-derived small-EVs by proteomic analysis to identify diagnostic biomarkers for CC. By profiling of small-EVs from two CC cell lines, 464 proteins were identified. Among these, approximately 90% were reported in the ExoCarta database [28]. Assignment of molecular function, biological process, cellular component, localization, and pathways revealed similar patterns to those observed in our previous studies [26]. This also corresponded with the current hypothesis for small-EV formation [29]. Figure 1 shows our strategy for selecting a diagnostic marker. We selected biomarker candidates that were cancer-related proteins in small-EVs produced by and exposed on the outer surface of cancer cells.

TSPAN1 is expressed in normal human tissues and carcinomas. Recently, TSPAN1 was also reported as a cancer-related protein [30, 31]. In several studies, overexpression of TSPAN1 was observed in liver cancer, prostate cancer, and gastric carcinoma. TSPAN1 plays a role in cell mitosis and causes abnormal cell differentiation. It was detected by RT-PCR in cervical cancer, lung cancer, squamous carcinoma, colon cancer, and breast cancer cells [30]. Wollscheid et al. evaluated *TSPAN1* mRNA levels by RT-PCR and TSPAN1 protein levels by immunohistochemistry in cervical cancer and found that the gene was expressed in grade III cervical intraepithelial neoplasia, cervical squamous cell carcinoma, and adenocarcinoma, particularly in all undifferentiated cervical carcinomas and adenocarcinomas [31]. They observed that TSPAN1 gene expression correlated with cell proliferation and suggested that it may be useful as a marker for cervical cancer prognosis.

In this study, a new strategy was established for identifying small-EV markers for CC diagnosis. However, further studies are required to evaluate their diagnostic value in clinical scenarios and elucidate their biological role(s) in cancer progression and metastasis. We performed western blotting to confirm the expression of candidate proteins in small-EVs from CC cells and normal colon cells and confirmed that SLC1A5 and LGALS3BP were differentially expressed between small-EVs and cells. However, previous studies reported that specific sorting of small-EV proteins can lead to changes in their composition [32, 33]. Expression levels of candidate proteins in small-EVs from the plasma of CC patients were confirmed by western blotting. We isolated small-EVs using ExoQuick solution. This method was fast and isolated small-EVs using only a small volume of plasma. However, recent studies reported that in current precipitation protocols, such as ExoQuick, small-EVs from cells and plasma were co-isolated with serum proteins [34]. Therefore, we used an optimized ExoQuick method to eliminate contaminants, such as albumin [25]. Recently, efforts have been undertaken to isolate small-EVs using more effective methods such as size-exclusion chromatography and tangential flow filtration [34, 35]. Western blot analysis of 12 clinical samples revealed that TSPAN1 is a potent biomarker for CC diagnosis. Notably, the expression level of TSPAN1 was significantly higher in CC patients than in HCs. This result was validated by ELISA for 67 clinical samples.

TSPAN1-positive EVs showed diagnostic potential for CC with high sensitivity (75.7%). In contrast, the CC biomarkers CEA and CA19–9 are currently adjunctively used for diagnosis and monitoring because of their low sensitivity [36, 37]. These results suggest that using TSPAN1-positive EVs or their combination with CEA or CA19–9 can increase the efficacy of diagnosis and that this strategy for discovering small-EV markers for cancer diagnosis is effective.

## Conclusions

In conclusion, this study demonstrated that TSPAN1 was abundantly present in small-EVs from two CC cell lines. Further, analysis of small-EV proteins can be beneficial for identifying biofluid-based biomarkers for cancer diagnosis. Because cancer-derived small-EVs are found in body fluids such as the plasma and serum, TSPAN1 can act as a small-EV biomarker for advanced CC diagnosis.

## Additional files

Additional file 1: **Table S1.** Protein list of small-EVs from HCT-116 and HT-29 CC cell lines. (XLSX 45 kb)

Additional file 2: **Table S2.** Protein list of small-EVs from CRL1541 normal colon cell line. (XLSX 14 kb)

## Abbreviations

AUC: Area under ROC curve
CC: Colon cancer
CEA: Carcinoembryonic antigen
CLDN7: Claudin-7
ELISA: Enzyme-linked immunosorbent assay
FDR: False discovery rate
GPRC5A: Retinoic acid-induced protein 3
HC: Healthy control
IPI: International Protein Index
LGALS3BP: Galectin-3-binding protein
NTA: Nanoparticle tracking analysis
PANTHER: Protein Analysis Through Evolutionary and Relationship
ROC: Receiver operating characteristic
SLC1A5: Neutral amino acid transporter B(0)
Small-EV: Small extracellular vesicle
TEM: Transmission electron microscope
TSPAN1: Tetraspanin-1
